# Complementary foods in baby food pouches: position statement from the Nutrition Commission of the German Society for Pediatrics and Adolescent Medicine (DGKJ, e.V.)

**DOI:** 10.1186/s40348-019-0089-6

**Published:** 2019-03-06

**Authors:** Berthold Koletzko, Christoph Bührer, Regina Ensenauer, Frank Jochum, Hermann Kalhoff, Burkhard Lawrenz, Antje Körner, Walter Mihatsch, Silvia Rudloff, Klaus-Peter Zimmer

**Affiliations:** 1 Div. Metabolic and Nutritional Medicine, Dept. of Pediatrics, Dr. von Hauner Children’s Hospital, University Hospital, LMU - Ludwig-Maximilians-Universität Munich, Munich, Germany; 2 Clinical Center for Neonatology, Charité Universitätsmedizin Berlin, Berlin, Germany; 3 Clinical Center for Paediatrics, Neonatology and Paediatric Cardiology, Center for Paediatrics and Adolescent Medicine, University Medical Center Düsseldorf, Research Institute of Child Nutrition, Max Rubner-Institut Karlsruhe, Düsseldorf, Germany; 4 Waldkrankenhaus Protestant Hospital Berlin Spandau, Berlin, Germany; 5 Hospital for Paediatrics and Adolescent Medicine Dortmund, Dortmund, Germany; 6 Private Practice for Paediatrics and Adolescent Medicine, Arnsberg, Germany; 7 Paediatric Research Center, Clinical Center and Polyclinic for Paediatrics and Adolescent Medicine, Department for Gynecology and Paediatrics, University Medical Center Leipzig, Leipzig, Germany; 8 Children’s Hospital Heliosclinic Pforzheim, Pforzheim, Germany; 9 Center for Paediatrics and Adolescent Medicine, Justus-Liebig-University Gießen, Giessen, Germany

**Keywords:** Complementary feeding, Sugars, Energy density, Nutrient requirements, Eating behavior, Parent-child interaction, Infant feeding

## Abstract

Pureed complementary feeding products packed in squeezable plastic pouches, usually with a spout and a screw cap, have been increasingly marketed. The Committee on Nutrition recommends that infants and young children should not suck pureed or liquid complementary foods from baby food pouches. Complementary foods should be offered with a spoon or should be fed as finger foods. Infants and young children should be given the opportunity to get to know a variety of foods and food textures including pieces of foods, supported by responsive feeding between the child and their parents or caregivers. Complementary foods marketed in baby food pouches often have a high energy density and are predominantly extremely high in sugar content, with up to almost 90% of the total energy content. Regular consumption bears the risks of imbalanced nutrient provision and increased risks for dental caries and overweight. Complementary foods for infants and young children should have a balanced composition following the recommendations of the German Society of Pediatrics and Adolescent Medicine (DGKJ) and should contain only limited amounts of sugar. We discourage the feeding of pureed complementary foods from baby food pouches.

**Electronic supplementary material:**

The online version of this article (10.1186/s40348-019-0089-6) contains supplementary material, which is available to authorized users.

Breastfeeding is the optimal form of infant feeding, with numerous health benefits for mother and child [1–3]. Between the beginning of the fifth and the beginning of the seventh month of life, complementary foods should be introduced in addition to breastfeeding. Complementary foods provide additional energy and critical nutrients such as iron, zinc, iodine, B vitamins, and long-chain polyunsaturated fatty acids (LC-PUFA) to support normal growth and development [1–3]. Complementary foods, including homemade or commercially available complementary foods for infants and young children, can be offered.

In recent years, there has been a rapid and increasing number and variety of pureed foods for infants and young children available on the market which are packed in compressible plastic bags and are usually equipped with a spout and a screw cap (so-called baby food pouches) [4]. Some products even have a spoon that can be screwed on and refilled directly from the baby food pouch. Based on the total volume, these products are often twice as expensive as conventional baby food fruit jars. However, from the point of view of many parents, baby food pouches offer a simple and convenient approach to complementary feeding. After unscrewing the screw cap, the food pouch contents can be squeezed directly into the mouth of the infant or the young child, or the child can suck or drink the contents from the food pouch spout, in the case of low-viscosity products like “smoothies.” In this way, mealtimes at home or on the road can be quick and easy. This type of complementary feeding has become very popular. Even reusable baby food pouches are sold, which allow infant feeding with pureed homemade complementary foods.

This type of complementary feeding may seem attractive for parents because it is perceived as time-saving and convenient; however, it raises serious concerns [5, 6]. The complementary feeding period is not only important for the provision of nutrients but it also serves as a time of transition from an exclusive milk diet to a diet of diversified family foods, with gradual learning of differentiated oropharyngeal movements and the development and shaping of eating behaviors [7, 8]. When infants receive complementary foods primarily through sucking foods from baby food pouches, this may delay or hinder learning to eat from a spoon or learning to eat finger foods [9]. The exploration of foods with the lips, tongue, and the hands as well as the practice of chewing can be impaired. Some observational studies indicate a potentially limited window of opportunity for favorable introduction of solid foods. For example, the delayed introduction of chunky foods after the age of about 9 to 10 months was associated with increased feeding difficulties and low intake of vegetables and fruits at later ages [10]. It, therefore, seems inadvisable to give infants and young children complementary foods which are mostly in semi-liquid or pureed consistency and which are predominantly consumed through sucking.

Moreover, if infants and young children are given a variety of textures and pieces of solid foods with a spoon or through feeding themselves by hand, it offers an opportunity for intense interaction between parents and children. This allows for mutual listening and dialog with the child, and for the monitoring and learning of hunger signals and sensitive responses (responsive feeding) [11, 12]. These opportunities can be wasted if children are allowed to suck complementary foods from a baby food pouch alone.

The typical composition of baby food pouches raises serious concerns. Product claims that are perceived positively by parents are often brought to the forefront, for example, natural or organic ingredients, the absence of artificial additives, gluten, or lactose, or the vegetarian or vegan characteristics of the product. However, many of these products have a high energy density, a very sweet taste, and a totally unbalanced nutritional composition with sugar contents that are too high. On the 13th and 14th of October 2018, a non-systematic and non-exhaustive internet search was carried out. The nutrient composition of 100 complementary food products offered in baby food pouches in Germany for infants and young children with an age indication: after the 4th month, from the 6th month, or from the 12th month of age were recorded (Additional file 1: Table S1). The search showed energy contents between 38 and 89 kcal/100 g product (median 60 kcal/100 g; 75th percentile 66 kcal/100 g; 90th percentile 75 kcal/100 g) and sugar contents between 40.0% and 88.9% of energy content (E%) (median 70.1 E%; 75th percentile 78.7 E%; 90th percentile 83.3 E%). Baby food pouches on offer contain predominantly sweet pureed fruit preparations. Even products with the claim of the content of cereals, vegetables, or dairy products are very sweet and have too high a sugar content. The 20 products with the highest energy content from sugar are shown in Table 1.

**Table 1:** Top 20 complementary foods sold in baby food pouches with the highest sugar and percent energy from sugar (g/100 g and % energy content). In a non-systematic internet research on 13th and 14th of October 2018, a total of 100 baby food pouches with the age recommendations: after the 4th month, from the 6th month onwards, and after the 12th month were recorded (see Additional file 1: Table S1)

Product	Energy kcal/100 g	Sugar g/100 g	Sugar % kcal
Sesame Street baby food pouch Elmo 100% apple, banana, and raspberry	63	14	88.9
Bebivita baby food pouch of pear raspberry in apple	49	10.5	85.7
Bebivita baby food pouch Squeeze Me! kiwi banana in apple	56	12	85.7
Hipp baby food pouch Super Hippis pomegranate acerola in apple raspberry	50	10.6	84.8
Erdbaer Freche Freunde baby food pouch 100% apple, pear, and passion fruit	52	11	84.6
Hipp baby food pouch Hippis peach in apple mango	55	11.5	83.6
Holle Baby food pouch apple and mango	58	12.1	83.4
MOGLi baby food pouch fruit drink banana rhubarb raspberry	71	14.8	83.4
Hipp baby food pouch Hippis wild berries in apple peach	49	10.2	83.3
Hipp baby food pouch Hippis wild berries in apple peach	49	10.2	83.3
Holle baby food pouch apple with carrot and parsnip	49	10.2	83.3
Erdbaer Freche Freunde baby food pouch 100% apple, strawberry, blueberry, and raspberry	43	8.9	82.8
Alete baby food pouch dragon fire strawberry banana	65	13.4	82.5
Hipp baby food pouch smoothie mix blueberry in apple pear	54	11.1	82.2
Hipp baby food pouch smoothie mix red fruits in apple banana	61	12.5	82.0
Hipp baby food pouch Hippis strawberry banana in apple	54	10.9	80.7
Hipp baby food pouch Hippis strawberry banana in apple	54	10.9	80.7
Holle baby food pouch apple and banana	66	13.3	80.6
Erdbaer Freche Freunde baby food pouch 100% apple, banana, spinach, and cucumber	50	10	80.0
DM organic baby food pouch banana-orange-beetroot	60	12	80.0

Complementary food products, which consist exclusively or predominantly of sweet fruit preparations, are not recommended as meals [1–3]. They not only contribute to high sugar intakes, which are questionable for health, but they also do not provide relevant quantities of critical nutrients that should be provided in addition to breastfeeding, in particular iron, zinc, iodine, B vitamins, and long-chain polyunsaturated fatty acids (LC-PUFA). In order to enable the child to get to know a variety of aromas and flavors, fruit should be offered as part of fruit-cereal porridges or offered separately after a diverse meal made up of vegetables, meat, or fish.

Pureed foods with high sugar content tend to adhere to tooth surfaces more so than chewed foods with abrasive properties and may therefore present an increased risk of developing dental caries [13–15]. This is especially true when these products are sucked out of the packaging and expose tooth enamel over an extended period of time. The combination of very high sugar content and organic fruit acids suggests an additional increase in caries risk.

There is also an indication that children who are regularly exposed to very sweet foods may experience potential long-term effects on their taste preferences and food choices. In observational studies, a high intake of sweet foods and drinks early on is associated with a higher preference for sweet foods in later life [16]. This is undesirable because of the associations between high intakes of sweet foods and the increased risk for dental caries, obesity, and associated non-communicable diseases [17].

For parents, giving a baby food pouch to their child with pureed fruit may seem equivalent to giving fresh fruit, but this is not the case. The high energy density in many complementary food products sold in baby food pouches is far higher than the typical energy density of fresh fruit (for example, 54 kcal/100 g apple). The extremely high sugar content in most products makes them unsuitable for feeding infants and young children. The high sugar content seems to come from the preferred use of very sweet fruit varieties and the addition of fruit juice concentrates, such as apple juice concentrate or grape juice concentrate or concentrated fruit preparations. Typically, the majority or even all of the sugar content comes from the fruit preparation used and not from added sugars, so that even products with extremely high sugar are labeled “no sugar added.” This claim may falsely create the impression with families that products are low in sugar, which may also lead to other family members, including siblings, to more regularly consume products. Not only “added sugar” but, above all, the total sugar content is responsible for undesirable effects on child health, such as adverse metabolic effects, high insulin secretion, increased risk for tooth decay, obesity, and other diseases.

The combination of a high energy density of these extremely sugar-rich products together with the ease of absorption by sucking the pureed product can result in a much higher energy and sugar intake—predominantly in the form of fructose—in a short time frame when compared to chewing and swallowing pieces of fruit. The very high sugar intake from a meal of pureed fruit preparation can be expected to significantly increase blood sugar and insulin levels compared to the consumption of fresh fruit. This is because the matrix of an intact fruit will usually lead to a slower sugar absorption than with a pureed preparation. For example, mashed potatoes had a much higher glycemic index (83) compared to cooked potatoes (49) [18]. A high glycemic index and a high glycemic load stimulate increased insulin secretion in children and can promote undesirable high weight gain and increased body fat deposition [19]. When regularly feeding infants and young children with fruit purees from baby food pouches, an increased risk of overfeeding and excessive weight gain must be expected, which is associated with a significantly increased risk for later obesity [20–22]. A high habitual sugar intake has also been associated with an increased, obesity-associated risk of cancer [23]. The birth cohort study Project VIVA in New England showed poorer cognitive performance (Kaufman Brief Intelligence Test II) with a high sugar intake in pregnancy and early childhood at 3–8 years of age [24].

Different fruits differ in their sugar contents and composition. In general, however, fruits are rich in fructose, and they also contain variable amounts of glucose and sucrose [25]. In many of the pureed fruit preparations, the fructose content is further increased, for example, by the addition of apple juice concentrate. With a high fructose intake from pureed fruit preparations, adverse metabolic effects are to be expected. A high fructose intake promotes de novo lipogenesis, fatty liver, and the occurrence of non-alcoholic fatty liver disease [26–28]. An important role of fructose intake is also supported by the findings of a study of 302 children with fructose malabsorption, who could only receive a limited amount of fructose and had a significantly lower frequency of obesity (2.3%) compared to a control group of children of the same age without limited fructose intake (6.1%) [29]. An increased risk for asthma has also been reported to be induced by fructose [30]. Although further investigations are needed to elucidate the pathophysiological and health effects of the various dietary sugars [31], currently available data suggest that sugar intake should ideally be below 5% of energy intake and in any case not exceed 10% of energy intake [17, 32].

Some baby food pouches have small screw caps as prescribed by the requirements of the European safety regulations for toys (EN 71), which are not legally binding for food packaging. However, there is a potential hazard for infants and young children due to the possibility of choking on small caps.

## Conclusion

Infants and toddlers should not suck pureed or liquid foods out of baby food pouches. Much more, infants and young children should be given the opportunity to get to know a variety of foods through spoon-feeding and by eating finger foods by hand. This should be supported through responsive feeding between the parent or caregiver and the child. It is recommended to feed home-prepared, balanced composite foods with a low sugar content and high content of vital nutrients such as iron, zinc, iodine, B vitamins, and long-chain polyunsaturated fatty acids, according to current recommendations [1–3]. These foods should offer a variety of taste and textures. If commercial complementary foods are used, they should be comprised and selected according to the same principles. Industrial fruit purees should not replace entire meals, but only be part of a meal (for example, in a fruit-cereal porridge) or be offered as a complement to a meal. Giving complementary foods in the form of drinks is not recommended. Pureed foods should be fed with a spoon and not be sucked out of food pouches.

## Additional file

Additional file 1: **Table S1.** Sugar and energy content (g/100 g product and % of energy content) in 100 complementary foods marketed as baby food pouches with the age recommendations: after the 4th month, from 6th month, and from the 12th month, sorted by sugar content (% of energy). Data from a non-systematic internet search on 13th and 14th of October 2018. (DOCX 31 kb)

## Abbreviations

DGKJ: German Association for Pediatrics and Adolescent Medicine
EN 71: European standard specification 71, safety regulations for toys
LC-PUFA: Long-chain polyunsaturated fatty acids
LMU: Ludwig-Maximilians-Universität München
