# Supplementary material for: Complementary foods in baby food pouches: position statement from the Nutrition Commission of the German Society for Pediatrics and Adolescent Medicine (DGKJ, e.V.)
Source: Mol Cell Pediatr. 2019 Mar 6;6:2. doi: 10.1186/s40348-019-0089-6 (PMC6403273; doi:10.1186/s40348-019-0089-6)
Supplement: Supplementary file 1 — Table S1. Sugar and energy content (g/100 g product and % of energy content) in 100 complementary foods marketed as baby food pouches with the age recommendations: after the 4th month, from 6th month, and from the 12th month, sorted by sugar content (% of energy). Data from a non-systematic internet search on 13th and 14th of October 2018. (DOCX 31 kb) [file 40348_2019_89_MOESM1_ESM.docx]

Additional file

**Complementary foods in baby food pouches**

Position Statement from the Nutrition Commission of the German Society for Paediatrics and Adolescent Medicine (DGKJ, e.V.)

**Table S1**: Sugar and energy content (g/100 g product and % of energy content) in 100 complementary foods marketed as baby food pouches with the age recommendations: after the 4^th^ month, from 6^th^ month and from the 12^th^ month, sorted by sugar content (% of energy). Data from a non-systematic internet search on 13^th^ and 14^th^ of October, 2018.

| **Product** | **Energy**  kcal/100 g | **Sugar**  g/100 g | **Sugar**  % kcal |
| --- | --- | --- | --- |
| Sesame Street baby food pouch Elmo 100 % Apple, Banana & Raspberry | 63 | 14 | 88.9 |
| Bebivita baby food pouch Pear-Raspberry in Apple | 49 | 10.5 | 85.7 |
| Bebivita baby food pouch Squeeze Me! Kiwi-Banana in Apple | 56 | 12 | 85.7 |
| Hipp baby food pouch Super Hippis Pomegranate-Acerola in Apple-Raspberry | 50 | 10.6 | 84.8 |
| Erdbaer Freche Freunde baby food pouch 100 % Apple, Pear & Passion Fruit | 52 | 11 | 84.6 |
| Hipp baby food pouch Hippis Peach in Apple Mango | 55 | 11.5 | 83.6 |
| Holle baby food pouch Apple & Mango | 58 | 12.1 | 83.4 |
| MOGLi baby food pouch fruit drink Banana Rhubarb Raspberry | 71 | 14.8 | 83.4 |
| Hipp baby food pouch Hippis Wild Berries in Apple Peach | 49 | 10.2 | 83.3 |
| Hipp baby food pouch Hippis Wild Berries in Apple Peach | 49 | 10.2 | 83.3 |
| Holle baby food pouch Apple with Carrot & Parsnip | 49 | 10.2 | 83.3 |
| Erdbaer Freche Freunde baby food pouch 100 % Apple, Strawberry, Blueberry & Raspberry | 43 | 8.9 | 82.8 |
| Alete baby food pouch Dragon Fire Strawberry Banana | 65 | 13.4 | 82.5 |
| Hipp baby food pouch Smoothie Mix Blueberry in Apple Pear | 54 | 11.1 | 82.2 |
| Hipp baby food pouch Smoothie Mix Red Fruits in Apple Banana | 61 | 12.5 | 82.0 |
| Hipp baby food pouch Hippis Strawberry Banana in Apple | 54 | 10.9 | 80.7 |
| Holle baby food pouch Apple & Banana | 66 | 13.3 | 80.6 |
| Erdbaer Freche Freunde baby food pouch 100 % Apple, Banana, Spinach & Cucumber | 50 | 10 | 80.0 |
| DM organic baby food pouch Banana-Orange-Beetroot | 60 | 12 | 80.0 |
| Holle baby food pouch Apple with Strawberry | 53 | 10.6 | 80.0 |
| Sesame Street Baby food pouch Cookie Monster 100 % Pear, Apple, Plum & Cinnamon | 60 | 12 | 80.0 |
| Sesame Street Baby food pouch Grover 100 % Banana, Apple, Strawberry & Blueberry | 70 | 14 | 80.0 |
| Holle Baby food pouch Banana, Apple, Mango & Apricot | 73 | 14.4 | 78.9 |
| Fruchtbar baby food pouch Mango Peach Banana | 66 | 13 | 78.8 |
| Erdbaer Freche Freunde baby food pouch 100 % Apple, Mango & Peach | 61 | 12 | 78.7 |
| Holle baby food pouch Apple & Peach with Forest Berries | 52 | 10.2 | 78.5 |
| Erdbaer Freche Freunde baby food pouch 100 % Apple, Pear, Carrot & Pumpkin | 49 | 9.6 | 78.4 |
| Holle baby food pouch Apple & Banana with Pear | 65 | 12.7 | 78.2 |
| Babylove baby food pouch Apple Mango in Peach with Oats | 54 | 10.5 | 77.8 |
| Fruchtbar baby food pouch Pineapple-Mandarin-Mango-Apple-Banana | 62 | 12 | 77.4 |
| Fruchtbar baby food pouch Watermelon, Strawberry, Apple, Pear & Rice | 63 | 12 | 76.2 |
| MOGLi Baby food pouch fruit drink Apple, Orange + Carrot | 47 | 8.9 | 75.7 |
| Fruchtbar baby food pouch Mango, Orange, Coconut & Banana | 74 | 14 | 75.7 |
| Hipp baby food pouch Hippis Apple Pear Banana | 60 | 11.3 | 75.3 |
| Fruchtbar baby food pouch KiBa Beat Cherry, Banana | 85 | 16 | 75.3 |
| Holle baby food pouch Apple & Pear | 59 | 11.1 | 75.3 |
| Fruchtbar baby food pouch Guava Papaya Mango Banana | 80 | 15 | 75.0 |
| Hipp baby food pouch Hippis Mango Passion Fruit in Pear Apple | 56 | 10.5 | 75.0 |
| MOGLi baby food pouch fruit drink Apricot Banana Quince | 63 | 11.8 | 74.9 |
| Fruchtbar baby food pouch Raspberry, Apple, Wholegrain | 75 | 14 | 74.7 |
| Bebivita baby food pouch Squeeze Me! Plum Cassis in Pear Banana | 62 | 11.5 | 74.2 |
| Babylove baby food pouch Watermelon & Banana in Apple | 59 | 10.8 | 73.2 |
| Erdbaer baby food pouch 100 % Apple, Pear & Cinnamon | 52 | 9.5 | 73.1 |
| Babylove baby food pouch Mango & Papaya in Apple | 57 | 10.4 | 73.0 |
| Holly baby food pouch Pear with Apple & Spinach | 55 | 10 | 72.7 |
| DM organic Apple Mango in a baby food pouch | 59 | 10.7 | 72.5 |
| Pumpkin Organics baby food pouch FREUDE Carrot Pear Spinach Dried Plum Peas | 48 | 8.7 | 72.5 |
| Holle baby food pouch Carrot, Mango, Banana & Pear | 51 | 9.2 | 72.2 |
| Babylove baby food pouch Mango & Banana in Apple with Carrot | 59 | 10.6 | 71.9 |
| Holle baby food pouch Pear & Banana with Kiwi | 68 | 12.2 | 71.8 |
| Babylove baby food pouch Peach & Banana in Apple | 58 | 10.4 | 71.7 |
| Fruchtbar baby food pouch Strawberry Apple | 51 | 9.1 | 71.4 |
| MOGLi baby food pouch fruit drink Apple Banana Strawberry | 60 | 10.7 | 71.3 |
| Erdbaer Freche Freunde Baby food pouch Apple, Pear, Blueberry & Spelt | 62 | 11 | 71.0 |
| Erdbaer Freche Freunde Baby food pouch Banana, Erdbeere & Quinoa | 80 | 14 | 70.0 |
| Hipp baby food pouch Hippis Kiwi in Pear Banana | 61 | 10.6 | 69.5 |
| DM Organic baby food pouch Pear-Strawberry in Apple | 57 | 9.9 | 69.5 |
| Babylove baby food pouch Watermelon & Banana in Apple | 53 | 9.2 | 69.4 |
| Erdbaer Freche Freunde Baby food pouch 100 % Apple, Banana, Pineapple & Coconut | 75 | 13 | 69.3 |
| Pumpkin Organics baby food pouch SONNIG Pumpkin Sweet Potato Pear Banana Blueberry | 45 | 7.8 | 69.3 |
| Fruchtbar baby food pouch Apple Forrest Fruit | 52 | 9 | 69.2 |
| Babylove baby food pouch Kiwi & Banana in Pear | 64 | 10.8 | 67.5 |
| Fruchtbar baby food pouch of Blueberry, Banana, Rice | 89 | 15 | 67.4 |
| MOGLi baby food pouch fruit drink Banana Guava Mango | 76 | 12.8 | 67.4 |
| Earth Bear Freche Freunde 100 % Pear, Banana, Orange & Vanilla | 66 | 10.9 | 66.1 |
| Hipp baby food pouch Hippis Raspberry Grape in Apple Banana with Oats | 70 | 11.5 | 65.7 |
| Holle Baby food pouch Pear & Apricot | 60 | 9.8 | 65.3 |
| Fruchtbar squeeze Pomegranate, Strawberry, Peach, Banana & Rice | 54 | 8.8 | 65.2 |
| Holle Baby food pouch Peach, Apricot & Banana with Spelt | 62 | 10.1 | 65.2 |
| Holle Baby food pouch Pear, Peach & Raspberry with Spelt | 59 | 9.6 | 65.1 |
| MOGLi Baby food pouch fruit drink Plum, Pear + Quince | 53 | 8.6 | 64.9 |
| Bebivita 1 Year Apple Banana Raspberry Cookies | 76 | 12.2 | 64.2 |
| Erdbaer Freche Freunde Baby food pouch 100 % Apple, Beetroot, Strawberry & Raspberry | 53 | 8.5 | 64.2 |
| Babylove baby food pouch Apple & Rosehip in Pear | 68 | 10.8 | 63.5 |
| Bebivita baby food pouch Squeeze me! Apple Peach Mango with Cookies | 72 | 11.4 | 63.3 |
| Pumpkin Organics baby food pouch TOP Carrot Water Apple Spinach Lentils | 42 | 6.5 | 61.9 |
| Holly Babyfood Baby food pouch Pear, Apple & Blueberry with Oats | 66 | 10.2 | 61.8 |
| MOGLi baby food pouch fruit drink Apple Blueberry Raspberry | 60 | 9.2 | 61.3 |
| Babylove baby food pouch Kiwi Pear with Peas | 60 | 9 | 60.0 |
| Hipp baby food pouch Hippis Peach Banana in Apple with Cookies | 75 | 11.2 | 59.7 |
| Fruchtbar baby food pouch Pear, Apple, Millet | 67 | 10 | 59.7 |
| Fruchtbar baby food pouch Pink Carrot Cherry Apple Millet | 62 | 9.2 | 59.4 |
| Erdbaer Freche Freunde Baby food pouch 100 % Apple, Banana & Raspberry | 77 | 11.4 | 59.2 |
| Babylove baby food pouch Bananas & Pineapple in Apple with Coconut Milk | 72 | 10.6 | 58.9 |
| Fruchtbar baby food pouch Raspberry Blueberry Grape Banana Apple | 75 | 11 | 58.7 |
| Pumpkin Organics baby food pouch WONNE Pumpkin Carrot Apple Banana Peach | 38 | 5.5 | 57.9 |
| Babylove baby food pouch Apple and Apricot Yoghurt with Quinoa | 58 | 8.2 | 56.6 |
| DM Organic baby food pouch Apple Banana with Spelt | 60 | 8.4 | 56.0 |
| Pumpkin Organics baby food pouch SPASS Carrot Pear Sweet Potato Broccoli | 39 | 5.3 | 54.4 |
| Pumpkin Organics baby food pouch GENUSS Sweet Potato Apple Spinach Corn Peas | 52 | 7 | 53.8 |
| MOGLi baby food pouch fruit drink Guava, Pineapple + Coconut | 68 | 9.1 | 53.5 |
| Fruchtbar baby food pouch Kiwi Strawberry Pear Banana Cookies | 77 | 10 | 51.9 |
| Erdbaer Freche Freunde Baby food pouch 100 % Apple, Sweet Potato & Mandarin | 71 | 9.2 | 51.8 |
| Babylove baby food pouch Strawberry Banana in Apple with Muesli | 58 | 7.4 | 51.0 |
| DM organic baby food pouch Apple-Banana-Strawberry with Oats | 59 | 7.5 | 50.8 |
| Pumpkin Organics baby food pouch HAPPY Apple Green Beans Spinach Kiwi | 40 | 5 | 50.0 |
| Pumpkin Organics baby food pouch ZAUBER Banana Carrot Broccoli Quinoa Blueberry | 52 | 6 | 46.2 |
| Erdbaer Freche Freunde Baby food pouch Strawberry & Raspberry in Yoghurt | 65 | 7.2 | 44.3 |
| Holle Baby food pouch Carrot & Sweet Potato with Peas | 43 | 4.3 | 40.0 |
